# Predictors of long-term disability in multiple sclerosis patients using routine magnetic resonance imaging data: A 15-year retrospective study

**DOI:** 10.1177/19714009221150853

**Published:** 2023-02-06

**Authors:** Amjad Altokhis, Abdulmajeed Alotaibi, Paul Morgan, Radu Tanasescu, Nikos Evangelou

**Affiliations:** 1 Mental Health and Clinical Neurosciences Academic Unit, School of Medicine, 6123 University of Nottingham, Nottingham, UK; 2 Clinical Neurology, Queen’s Medical Centre, 6123 University of Nottingham, Nottingham, UK; 3 Department of Radiological Sciences, School of Health and Rehabilitation Sciences, 112893 Princess Nourah Bint Abdulrahman University, Riyadh, Saudi Arabia; 4 Department of Radiological Sciences, School of Applied Medical Sciences, 48149 King Saud Bin Abdul-Aziz University for Health Sciences, Riyadh, Saudi Arabia; 5 Sir Peter Mansfield Imaging Centre, School of Medicine, 6123 University of Nottingham, Nottingham, UK; 6 NIHR Nottingham Biomedical Research Centre, Queen’s Medical Centre, 6123 University of Nottingham, Nottingham, UK; 7 Medical Physics and Clinical Engineering, 9820 Nottingham University Hospitals NHS Trust, Nottingham, UK

**Keywords:** Multiple sclerosis, magnetic resonance imaging, white matter lesion, lesion load, volume

## Abstract

**Introduction:**

Early identification of patients at high risk of progression could help with a personalised treatment strategy. Magnetic resonance imaging (MRI) measures have been proposed to predict long-term disability in multiple sclerosis (MS), but a reliable predictor that can be easily implemented clinically is still needed.

**Aim:**

Assess MRI measures during the first 5 years of the MS disease course for the ability to predict progression at 10+ years.

**Methods:**

Eighty-two MS patients (53 females), with ≥10 years of clinical follow-up and having two MRI scans, were included. Clinical data were obtained at baseline, follow-up and at ≥10 years. White matter lesion (WML) counts and volumes, and four linear brain sizes were measured on T2/FLAIR ‘Fluid-Attenuated-Inversion-Recovery’ and T1-weighted images.

**Results:**

Baseline and follow-up inter-caudate diameter (ICD) and third ventricular width (TVW) measures correlated positively with Expanded Disability Status Scale, ≥10 or more of WMLs showed a high sensitivity in predicting progression, at ≥10 years. A steeper rate of lesion volume increase was observed in subjects converting to secondary progressive MS. The sensitivity and specificity of both ICD and TVW, to predict disability at ≥10 years were 60% and 64%, respectively.

**Conclusion:**

Despite advances in brain imaging and computerised volumetric analysis, ICD and TVW remain relevant as they are simple, fast and have the potential in predicting long-term disability. However, in this study, despite the statistical significance of these measures, the clinical utility is still not reliable.

## Introduction

Multiple sclerosis (MS) is a chronic inflammatory, demyelinating and neurodegenerative disease of the central nervous system (CNS).^1^ The early identification of MS high-risk patients with a greater disability is crucial and it would be effective for determining an early personalised treatment strategy. Studies have shown that more than half of MS patients are likely to develop a significant disability after 15–30 years;^2–4^ however, some cohorts showed that many maintained a mild disease state.

In a largely untreated cohort of people with clinically isolated syndrome (CIS), it has been found that up to 42% of patients remained fully ambulatory (EDSS ≤3.5) 30 years later, while 58% developed disability and may have benefitted from early treatments.^2^ It has also been reported that patients who accumulated lesions over the first five years of their disease were more likely to develop secondary progressive multiple sclerosis (SPMS) 20 years later,^2^ especially when 10 or more lesions are present at the baseline scan.^4^ It is therefore important to examine if there is an easily applicable measurement that could predict long-term disability in an era the disease-modifying therapies (DMTs) have been used.^5^ EDSS 4 and EDSS 6 are routinely used as disease progression cut-offs because EDSS 4 is the first point in the scale at which walking is limited/restricted, and EDSS 6 is the first point at which bilateral walking aids are required.

Despite advances in brain imaging and computerised volumetric analysis, manual brain measurements of the lesions and linear brain atrophy remain relevant as they are simple, fast, and can be performed on non-digitised data. In addition, for brain atrophy measures, linear two-dimensional methods do not require extensive training or expensive and time-intensive computer software necessary for complex quantitative and volumetric analyses. Furthermore, imaging protocols do not always include three-dimensional brain imaging (required for voxel-based morphometry) due to time constraints while two-dimensional scans have shorter acquisition times and are easily available. For these reasons, simple linear measurements are applicable to clinical practice, especially in regions with no access to advanced imaging technology.

Different MRI predictors of MS disability which can be easily implemented in clinics have been proposed.^6–9^ To date, these predictors have not been approved to be implemented in clinical practice. Predictors include white matter lesions (WMLs) accumulation^10,11^ and linear measurements of brain atrophy. Amongst the many proposed linear measures of brain atrophy, the most widely reported are third ventricular width (TVW),^3^ medullary width (MEDW)^3,12^ corpus callosum index (CCI)^13^ and inter-caudate diameter (ICD).^12^ These measurements are established and practical techniques that correlate with long-term disability in MS.^14,15^ However, the reliability and strength to use these measures in clinics are yet to be confirmed in clinical MS cohort.

This study aims to assess the role and reliability of measures, that can feasibly be implemented within a clinical setting in the first 5 years of the disease, for predicting long-term (>10 years) outcomes. This assessment is needed to facilitate and implement reliable and practical MRI metrics in clinics.

## Material and methods

### Patient selection

This is a retrospective study from the MS clinic database at the Queen’s Medical Centre in Nottingham. In this study, data collected up to January 2021 were included. A total number of 3801 patients with different MS subtypes were registered with the clinic with the date of disease onset ≤2011. Inclusion criteria were (1) baseline brain MRI scan in 2011 or earlier (2) follow-up scan acquired between 4–6 years from the baseline scan (3) both sets of scans had a T2-FLAIR (fluid-attenuated inversion recovery) and T1 sequences of brain MRI (4) Patients with confirmed diagnosis of MS on the McDonald criteria 2010 (5) consented to have their MRI and clinical data used for research purposes (6) Primary progressive MS (PPMS) patients were excluded due to the different nature of the disease.

Patients’ demographic data were captured at baseline and follow-up MRI, and clinical data were extracted from the clinical notes. Data include age, sex, date of MS onset, date of MS diagnosis, MS subtypes, EDSS, date reached EDSS 4 and 6 if applicable and details of DMTs used. The number and volume of T2-FlAIR lesions at baseline and follow-up scans were measured by a researcher blind to their clinical characteristics.

Disability was evaluated according to the EDSS at three times; baseline, follow-up (4–6 years interval), and last visit (≥10 years). Disability milestones were defined as reaching an EDSS score ≥ 4.0 or 6.0, respectively, in addition to disease conversion to SPMS. A confirmed diagnosis of SPMS was defined by steady progression rather than relapse as the major cause of increasing disability in the preceding 2 years; and evidence of progression - either an increase of at least one point in EDSS or clinical documentation of increasing disability in patient notes. The follow-up duration was computed as the time between the date of the disease onset and the date of the most recent last visit.

We divided treatments in high-efficacy treatments (HET): Tysabri (natalizumab), Lemtrada (alemtuzumab, Campath), Ocrevus (ocrelizumab); and non-HET: beta-Interferons, Copaxone (glatiramer acetate), Aubagio (teriflunomide), Tecfidera (dimethyl fumarate), Gilenya (Fingolimod), Mavenclad (Cladribine).^16^

### MRI protocol

Clinical brain MRI scans were performed as part of the service provided at the MS clinic at the Queen’s Medical Centre using 1.5T and 3T MRI. These scans included sagittal T2-FLAIR and T1-weighted spin-echo. All the sequences were obtained using a contiguous 3–5 mm slice thickness covering the whole brain. Having a normal brain MRI scan was not an exclusion criterion, since patients were included based solely on their clinical diagnosis (i.e., patients with normal baseline brain scans were included).

### Image analysis

#### Lesions and atrophy

All measurements were performed using 3D slicer version 4.13.0 (https://www.slicer.org). Inter-rater and intra-rater reliability statistics were provided in (S.1). Lesions with long axis diameter ≥ 3 mm were included to satisfy diagnostic criteria.^17^ The total number and volume of WML for each scan were calculated. The level tracing segmentation tool on a 3D slicer was used to trace around each lesion on the scan, and volumes were recorded in cm^3^. The measurements for brain atrophy have been reported previously for TVW,^3,12^ MEDW,^3^ CCI^13^ and ICD.^12^ Further details about the segmentation are in (S.2).

#### Statistical analysis

Pearson’s correlation coefficient was used to assess the relationship between EDSS at > 10 years/change in EDSS and the six brain MRI metrics (lesion count, lesion volume, TVW, CCI, ICD, MEDW) at baseline, follow-up and changes in MRI parameters were computed as an annualized measure (e.g., CCI change/y). To assess the extent to which MRI brain metrics can be used to predict worsening disability over 10 years, we performed a simple linear regression analysis. The EDSS scores at 10 years were set as the dependent variable while MRI predictors (lesion count, lesion volume, TVW, CCI, ICD, MEDW) at baseline or follow-up were set as the independent explanatory variable, including a factor variable with three levels (HET, non-HET and untreated) in the model. Linear regression is particularly interpretable; however, EDSS is an ordered categorical variable with a scale (0–10). Therefore, ordinal regression analysis was also performed to check similar findings. The t-test tests were used to compare EDSS and MRI predictors between baseline, follow-up, and yearly change.

For the binary logistic regression, the EDSS models were EDSS ≥ 4 or 6 (dependent variables), while MRI predictors (lesion count, lesion volume, TVW, CCI, ICD, MEDW) at baseline or follow-up were set as the independent explanatory variable, including a factor variable with three levels (HET, non-HET and untreated) in the model. We calculated the sensitivity and specificity of different cut-offs of T2 lesions count. Inter and intra-rater errors were assessed in a sample of 10 randomly chosen scans. Statistical analysis was undertaken with *Jamovi* (Version 1.6), and statistical significance was reported at *p* <0.05.

## Results

### Patient characteristics

**Figure 1.**
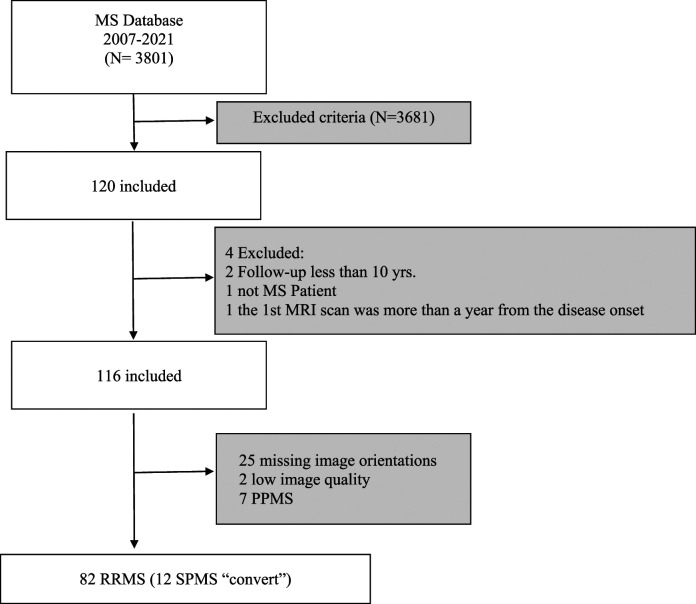
Flowchart illustrates the process of patient selection for this study.

Of the 3801 MS patients that were recorded, 82 RRMS patients were included based on the inclusion criteria (Figure 1). MRI brain scans of the 82 patients were 29 male and 53 female, with a mean age (±SD) of 35.4 (±10.3) years.

**Figure 2.**
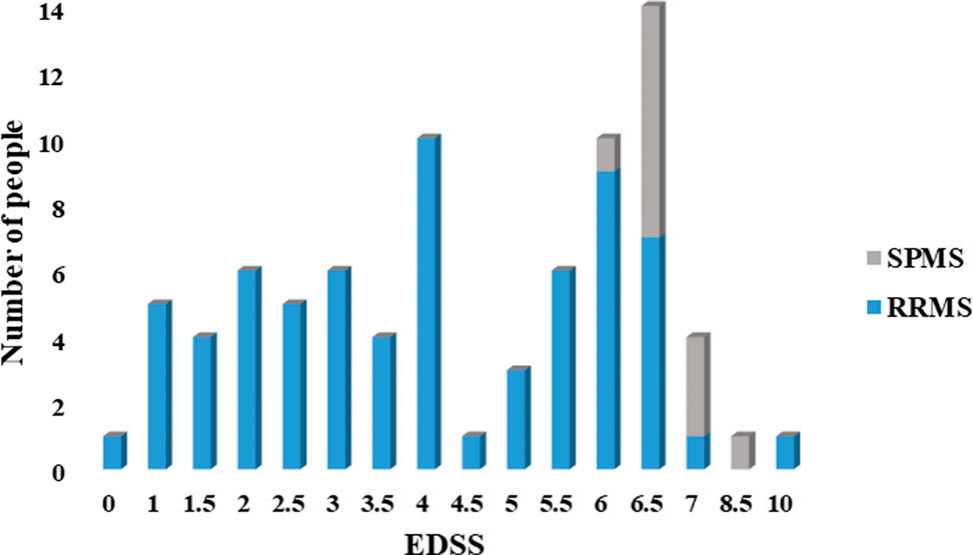
Expanding Disability status scale (EDSS) scores after more than 10 years of follow-up. EDSS were obtained from 82 patients at the last visit >10 years. An EDSS of 10 was assigned to those where Multiple sclerosis (MS) was known to contribute to death. *RRMS: Relapsing remitting multiple sclerosis; SPMS: Secondary progressive multiple sclerosis.

The mean clinical follow-up was 12.1 (±1.31) years, and the mean time between scans was 5.33 (±1) years. The baseline MRI scan was acquired with a median of 3 months (IQR 6) after disease onset. A wide range of physical disabilities encompassing all levels of the EDSS was seen across the cohort (Figure 2). EDSS worsened from a mean of 1.95 (±1.59) at baseline to 4.48 (±2.17) after 10 years (*p* <0.001). Out of 82 patients, 70 remained RRMS and 12 progressed to SPMS. During the follow-up, 51 RRMS patients reached EDSS 4 at the last visit while 31 patients reached EDSS 6. Two RRMS patients died and only one was due to MS.

Table 1 illustrates the characteristics of this cohort. The median time from the disease onset to DMTs initiation was 36 months (IQR 59.5). In terms of treatment, 23 patients received HET, 44 received non-HET and 15 patients were untreated. From the 67 treated patients, the mean duration of treatment was 90.6 months (SD 45.3) with a range of (2–168). On average, patients were treated with DMTs in 61% of their disease duration. The follow-up scan was requested as a standard procedure in 41 patients, or with new or worsening symptoms in 48 patients. Treatment with different levels of DMTs did not appear to contribute significantly to any of the models and thus were removed from the model.

**Table 1.** Demographic characteristics, clinical classification and data availability at each follow-up time points.

Characteristic	Baseline (*N* = 82)	Follow-up MRI (*N* = 82)	Last visit (*N* = 82)
Female sex, *n* (%)	53 (64.6%)	53 (64.6%)	53 (64.6%)
Age, mean (SD)	35.4 (10.3)	40.8 (10.2)	47.5 (10.1)
EDSS, median	1.5 (0–7)	3.25 (1–7.5)	4.25 (0–10)
Number DMTs treated, *n* (%)	0	51 (62.1%)	69 (84.1%)
Onset of treatment	—	2011 (2006–2016)	—
T2 lesion count	15.0 (0–58)	25.0 (2–99)	—
Total lesion volume (cm^3^)	1.48 (0–60.3)	4.44 (0.6–55.10)	—
Third ventricle (mm)	2.79 (1.27–7.31)	3.58 (1.69–8.85)	—
Corpus callosum (mm)	0.39 (0.23–0.48)	0.36 (0.20–0.44)	—
Medulla (mm)	8.19 (5.77–12.4)	7.72 (5.77–11.9)	—
Inter- caudate distance (mm)	13.5 (7.08–22.4)	15.2 (8.65–26.8)	—
Clinical follow-up: 12 (10–15) years	Time between scans: 5.33 (3.25–7.92) years		

All data presented as medians and ranges, unless otherwise stated, EDSS: Expanded Disease Duration Status Scale, DMTs: disease-modifying treatments.

The numbers of slices and slice thickness were variable, both between patients and between the first and second scans. Of the 164 scans analysed (first and follow-up MRIs), the number of slices ranged from 15 to 60 and slice thickness ranged from 3 to 5 mm. The mean number of slices was 26.7 (±5.94) and the mean slice thickness was 3.91 mm (±0.76).

### Baseline brain MRI and new lesions

The baseline MRI scans were normal in 1% of the patients; 30% had between 1–9 T2 WML, and 68% had more than 10 T2 WML. The new lesions and the yearly change in lesion counts are illustrated in Table 2.

**Table 2.** New lesions and the change rate in lesions on the follow-up MRI scan.

New lesions in the follow-up scan (4–6 years)	Number of patients
0–3	25
4–6	18
7–9	10
10–13	10
14–16	4
17–20	4
>21	14
Fewer lesions	4

Change in lesion count/year	Number of patients (%)
0–0.4	17 (20.7%)
0.41–0.8	16 (19.5%)
0.81–1	7 (8.5%)
1.1–2	15 (18.3%)
2.1–3	9 (11.10%)
3.1–4	4(4.9%)
4.1–5	5(6.1%)
5.1–6	3(3.7%)
6.1–7	2 (2.4%)
7.1–8	2 (2.4%)
9	1 (1.2%)
11	1 (1.2%)

## MRI predictors and long-term physical disability

### Correlations between MRI predictors and clinical disability

No correlations were found between WML counts and volume with clinical disability at both baseline and follow-up. Alternatively, some linear brain measurements showed a positive correlation with EDSS at the last visit. This included ICD (*r* = 0.25, *r* = 0.27, *p* < 0.01 at baseline and follow-up) and TVW (*r* = 0.28, *r* = 0.24, *p* < 0.01 at baseline and follow-up).

All correlations of MRI metrics with clinical disability are listed in Table 3. Considering the correlation between changes in EDSS from the follow-up scan, and the yearly change in MRI predictors, over the first 5 years, was only significant in the case of CCI, but with weak, positive correlations (*r* = 0.31, *r* = 0.30, *p* < 0.01).

**Table 3.** Correlations between MRI predictors and disease severity (EDSS).

Predictors	Pearson, (95% CI)
T2 lesions	
• BL Lesion count	r = 0.06, (−0.15, 0.27)
• FU lesion number	r = 0.08, (−0.13, 0.29)
• Change rate in lesion number	r = 0.08, (−0.13, 0.30)
• BL total lesion volume	r = 0.10, (−0.11, 0.31)
• FU total lesion volume	r = 0.08, (−0.13, 0.29)
• Change rate in total lesion volume	r = −0.09, (−0.22, 0.20)
Brain linear measurements	
• BL CCI	r = −0.09, (−0.30, 0.12)
• FU CCI	r = −0.04, (−0.25, 0.17)
• Change in CCI	r = 0.12, (−0.09, 0.33)
• BL MEDW	r = 0.007, (−0.21, 0.22)
• FU MEDW	r = −0.07, (−0.28, 0.14)
• Change rate in MEDW	r = −0.11, (−0.10, 0.32)
• BL ICD	r = 0.25, (0.03, 0.44)^a^
• FU ICD	r = 0.27, (0.01, 0.46)^a^
• Change rate in ICD	r = 0.14, (−0.07, 0.34)
• BL TVW	r = 0.28, (0.07, 0.47)^a^
• FU TVW	r = 0.24, (0.03, 0.43)^a^
• Change rate TVW	r = 0.22, (0.006, 0.42)^a^

Change in EDSS (from 1st MRI to last visit)	Pearson, (95% CI)
T2 lesions	
• BL Lesion count	r = −0.15, (−0.35, 0.06)
• FU lesion number	r = −0.09, (−0.30, 0.12)
• Change rate in lesion number	r = 0.14, (−0.20, 0.23)
• BL total lesion volume	r = −0.16, (−0.36, 0.05)
• FU total lesion volume	r = −0.12, (−0.24, 0.23)
• Change rate in total lesion volume	r = −0.04, (−0.25, 0.17)
Brain linear measurements	
• BL CCI	r = 0.18, (−0.03, 0.38)
• FU CCI	r = 0.31, (0.10, 0.49)^a^
• Change rate in CCI	r = 0.30, (0.09, 0.48)^a^
• BL MEDW	r = −0.07, (−0.14, 0.29)
• FU MEDW	r = −0.08, (−0.29, 0.13)
• Change rate in MEDW	r = −0.18, (−0.03, 0.38)
• BL ICD	r = 0.11, (−0.10, 0.32)
• FU ICD	r = 0.11, (−0.10, 0.32)
• Change rate in ICD	r = 0.03, (−0.18, 0.25)
• BL TVW	r = −0.007, (−0.14, 0.28)
• FU TVW	r = −0.08, (−0.13, 0.29)
• Change rate TVW	r = 0.08, (−0.13, 0.30)

BL: Baseline, FU: Follow-up, EDSS: Expanded Disability Status Score, CCI: Corpus callosum index, MEDW: medulla width, TVW: Third ventricular width.

^a^Significance at *p*-value of <0.05.

### T2 lesions and brain changes

As expected, the lesion count at the follow-up scan was greater than baseline (25 versus 15 lesions, *p* < 0.001), with an annual rate of new lesions of 1.11 lesion/year (*p* < 0.001). Similarly, the lesion volume was greater in the follow-up scan (1.48 versus 1.44 cm^3^, *p* < 0.001), with an annual lesion growth rate of 0.16 lesion/year (*p* < 0.001). Only 3 patients; 2 RRMS, and 1 SPMS did not have any new lesions, they had fewer lesions at the follow-up scan by 1 to 4 lesions.

Patients who remained RRMS or progressed to SPMS at the end of the observational period had similar medians of the baseline T2 lesion volume see Table 4. However, observing the pattern of T2 lesion volume during the follow-up scan showed a steeper rate of lesion volume increase in SPMS compared to RRMS over the first 4-6 years of the disease.

Baseline and follow-up brain linear measurements showed that MEDW and CCI had a greater mean at baseline, while ICD and TVW had a greater mean at the follow-up (Table 4). The median yearly changes TVW (3.12 mm/year, *p* < 0.001), ICD (0.35 mm/year, *p* < 0.001), MEDW (0.13 mm/year, *p* < 0.001), CCI (−0.005 mm/year, *p* < 0.001).

**Table 4.** White matter lesions counts and volume in relapsing-remitting and secondary-progressive MS patients.

MS subtypes	Baseline		Yearly change		Follow-up	
	Number	Volume	Number	Volume	Number	Volume
RRMS	16 (0-58)	2.44 (0-26.7)	1.06 (0-11.8)	0.15 (-6.38-9.63)	25.5 (2-99)	4.47 (0.60-31.4)
SPMS	13 (3-48)	2.34 (0.17-24)	1.22 (0.14 -9.71)	0.23 (-1.35 1.10)	24 (6-58)	4.44 (0.65 -25.8)

All data presented as medians and ranges RRMS: Relapsing-Remitting Multiple Sclerosis, SPMS: Secondary Progressive Multiple Sclerosis.

### EDSS at last visit (≥10 years)

The results from the linear regression showed that most of the predictors have positive associations, apart from CCI and MEDW which were not statistically significant (Table 5). Two predictors, TVW and ICD at both baseline and follow-up had statistically significant associations with long-term disability. For every unit increase in TVW at baseline, the value of disability score at the last visit was estimated to increase by a value of 0.45 mm and this association was highly statistically significant (*p* = 0.001). Linear regression may not be ideal for ordinal categorical variables such as EDSS, although it is easy to interpret, therefore we also performed ordinal regression and similar results were found (S.3).

### EDSS of 4.0 and 6.0 at 10 years

The risk of disability was more in patients with at least 10 baseline lesions. In such cases, 4 patients reached an EDSS 4 and 16 patients needed a cane within the first 6 years. These numbers rose to 60 and 30% respectively within the first 10 years of the disease. Fifty-six of the patients displayed ≥10 T2 WMLs on the baseline MRI. Of the 13 patients displaying 0 to 3 lesions at MRI baseline, 8 patients (61.53%) had an EDSS of at least 4, while six patients (23.0%) had an EDSS of at least 6. This percentage increased by 1% for EDSS 4 and 19.8% for EDSS 6 for patients with ≥10 lesions on the baseline (see Table 6).

**Table 5.** Linear regression for the predictors and EDSS.

Predictors	Coefficient (β)	95% CI
BL lesion count	0.009	(−0.02, 0.04)
FU lesion count	0.009	(−0.01, 0.03)
BL lesion volume	0.04	(−0.05, 0.13)
FU lesion volume	0.03	(−0.05, 0.11)
BL CCI	−4.39	(−15.06, 6.29)
FU CCI	−2.00	(−12.39, 8.39)
BL MEDW	0.01	(−0.37, 0.40)
FU MEDW	−0.14	(−0.56, 0.28)
BL ICD	0.18^a^	(0.02, 0.33)
FU ICD	0.16^a^	(0.03, 0.28)
BL TVW	0.45^a^	(0.12, 0.79)
FU TVW	0.30^a^	(0.03, 0.56)

BL: Baseline, FU: Follow-up, EDSS: Expanded disability status score, CC: Corpus callosum, MEDW: Medulla width, ICD: Inter-caudate diameter, TVW: Third ventricular width.

^a^Significance at *p*-value of <0.05.

Similar to the linear/ordinal regression findings, the binary logistic regression also showed that TVW at baseline could predict EDSS 4 and EDSS 6 after 10 years (Table 7). For EDSS ≥6, TVW at baseline/follow-up and ICD at baseline were significant predictors of disability while TVW at baseline was the only predictor for EDSS 4. In another word, the odds of TVW at follow-up is 0.75 higher with patients with EDSS ≥6.

**Table 6.** Baseline MRI lesion number and clinical status at 15 years.

	0–3 (*N* = 13)	4–9 (*N* = 13)	10+ (*N* = 56)
EDSS ≥4 at 15 years	8 (61.5%)	8 (61.5%)	35 (62.5%)
EDSS ≥6 at 15 years	3 (23.0%)	4 (30.7%)	24 (42.8%)

**Table 7.** Binary logistic regression for the predictors and EDSS ≥4 or EDSS ≥6.

Predictors	Odd ratio	95% CI	*p*-value
EDSS ≥4			
BL lesion count	0.98	(0.95–1.02)	0.39
FU Lesion count	0.99	(0.96–1.02)	0.60
BL lesion volume	0.92	(0.81–1.02)	0.16
FU Lesion volume	0.96	(0.88–1.05)	0.35
BL CCI	12.98	(4.6e-4–361645.2)	0.62
FU CCI	3.97	(0.0–499510.6)	0.85
BL MEDW	0.84	(1.25e-4–46171.8)	0.86
FU MEDW	2.40	(0.53–1.20)	0.27
BL ICD	0.89	(0.76–1.05)	0.16
FU ICD	0.95	(0.84–1.08)	0.48
BL TVW	0.67^a^	(0.45–1.01)	0.05^a^
FU TVW	0.90	(0.68–1.18)	0.44
EDSS ≥6			
BL lesion count	0.97	(0.94–1.01)	0.13
FU Lesion count	0.98	(0.96–1.01)	0.33
BL lesion volume	0.92	(0.83–1.02)	0.10
FU Lesion volume	0.95	(0.88–1.03)	0.24
BL CCI	7963.91	(0.27–2.32e + 8)	0.08
FU CCI	1165.00	0.06–2.15e + 7)	0.15
BL MEDW	1.02	(0.71–1.46)	0.90
FU MEDW	0.95	(0.65–1.41)	0.82
BL ICD	0.86^a^	(0.73–1.00)	0.05^a^
FU ICD	0.90	(0.79–1.02)	0.09
BL TVW	0.57^aa^	(0.38–0.84)	0.005^aa^
FU TVW	0.75^a^	(0.58–0.99)	0.04^a^

BL: baseline, FU: follow-up, EDSS: Expanded Disability Status Score, CC: corpus callosum, MEDW: medulla width, TVW: third ventricular width.

^a^Significance at *p*-value of <0.05.

### Aggressiveness MS: EDSS of 6.0 at 10 years

**Figure 3.**
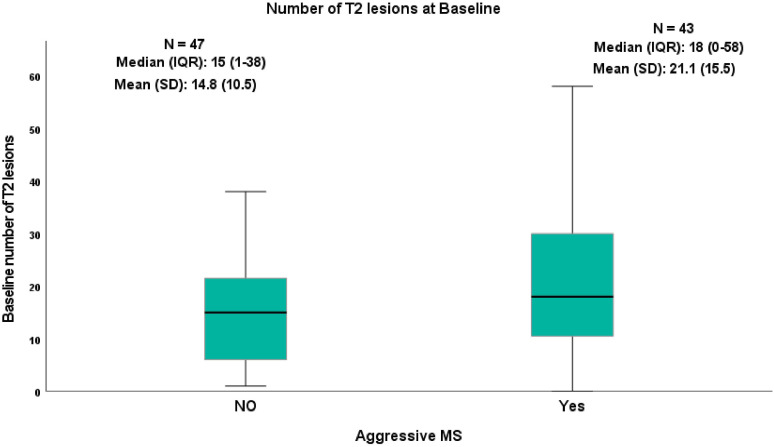
Number of T2 lesions at baseline in two MS groups, aggressive MS patients who reached EDSS ≥6 and EDSS <6.

As stated, aggressiveness was set as EDSS ≥6. The risk of having aggressive MS in the group of more than 10 lesions at baseline was 18% higher compared to patients with less than 10 lesions and this difference was highly statistically significant (*p* = 0.004). Furthermore, the median (IQR) number of baseline T2 lesions was 18 (0–58) in the aggressive group compared to 15 (1–38) in the non-aggressive group (Figure 3). In addition, the area under the curve was 0.60 (95% CI 0.48–0.72) which means poor prediction; the best cut point was 4 lesions at baseline with a high sensitivity of 88% but low specificity of 20%. A cut-point of ten lesions had better specificity of 43% but sensitivity was reduced by 74%.

ICD and TVW had similar values. A cut-point of 0.46 mm had the best specificity of ∼64% and sensitivity of ∼60 for both ICD and TVW, accuracy of 62% and AUC was 0.70 (95% CI 0.16–0.94) which is consider as acceptable predictors.

## Discussion

This study assesses the role and reliability of different MRI measures during the first 5 years of the disease, which can be easily used in routine clinics and could reliably predict MS disability.

The current study demonstrated that linear brain atrophy metrics related to ICD and TVW have an independent impact in predicting disability after 10 years but WML counts and volume showed no association with disability. However, the presence of 10 or more WML showed a high sensitivity in predicting disability.

More than 37% of our patients developed an aggressive MS (reaching an EDSS of 6 or more at 10 years). It is worth highlighting that in patients with an aggressive phenotype, baseline characteristics such as age and sex did not contribute in identifying patients at risk.

Contrary to previous findings,^10,18,19^ in our study, the counts and volume of WML were not associated with disability either at baseline or during the follow-up period. This could be due to the small sample size compared to other studies. Having said that, the correlation coefficient (R-value) was smaller compared to other studies and the should not be affected by study size.

 It is more likely that our results are to the patients included, which had definite MS,rather than CIS and by definition a more active disease with different lesions evolution characteristics. In a recent study including 548 placebo-treated RRMS patients, the multivariable analysis indicated EDSS score and T2 lesion load as factors that independently predict clinical progression. Nevertheless, these two variables taken together were able to account for only 3% of the probability to have an EDSS increase over follow-up time. Thus, confirming the limited value of these metrics in predicting disability changes in RRMS.^20^ Such a result is in line with several previous cross-sectional and longitudinal studies conducted on a smaller group of patients with different clinical characteristics, which have shown only a modest correlation between T2 and changes in disability.^21,22^ The presence of 10 or more of WMLs at baseline scan has attracted attention since the Barcelona group reported as being very predictive of how agressive the disease appears to be 10 years later.^23^ In our cohort, we did not replicate this finding. Although in the recent Barcelona study^4^ the median number of baseline T2 lesions in the aggressive group (EDSS 6 at 10 years) was higher compared to our cohort (17 vs 18). Again, the most likely explanation is that that they included CIS patients whereas we only included MS patients showed some merit to the use of this measure in progression prediction, which also has been reported previously.^24^ Furthermore, lesion load continues to increase in RRMS patients and the rate of lesion growth in those who develop SPMS is higher than those who remain RRMS, in line with a previous finding.^19^

Several reasons might explain the weak or absent association of WML and/or regional atrophy with clinical changes. This might be related to some technical limitations such as the difference in slice thickness and noise as manual measurements are generally more susceptible to it.

There are no previous studies investigating critical cut-off values for MRI volumetric predictors of disability, such as TVW. Therefore, a direct comparison of our findings with the existing literature’s cut-off values was not possible.

In the current study, the MEDW was measured as part of our dataset as a previous study showed that brainstem measures are sensitive to atrophy in MS^25^ and may act as a replacement for cervical spinal cord volume in predictive diagnostics.^3^ In another stud, MEDW measures were linearly correlated with disease severity in MS patients.^26^ However, in the current study, the similar metric of MEDW did not produce any statistical significance as a prognostic predictor. These findings could suggest that there is some independence of the MS pathology in these regions, as this lack of correlation between the brainstem and spinal cord measurements has been reported previously.^24^

Similarly, CCI was demonstrated in a previous study to be correlated to disease progression in MS patients but was not itself an independent predictor.^27^ Accordingly, CCI showed an annual decrease in patients following MS diagnosis, and the severity of CCI decrease was double in SPMS compared to RRMS patients with identifying a trend for a slower rate of CCI in patients using DMTs. Consistent with this line of thinking, the use of CCI as a measure of disease progression may not have been as statistically robust.

A high proportion of our patients received DMTs during the follow-up, and for the majority, there was a short time between the first clinical event and treatment initiation. Although only 62% of patients were using DMTs at clinical follow-up, there may have been a tendency for use of DMTs to be more common in patients with mild to aggressive symptoms. It was found that the treatment effect on disability progression was independently correlated with brain atrophy and the presence of active MRI lesions.^28^

This study has limitations that include the exclusion of a large subset of patients when patients with MS were not routinely scanned annually during the first years of their disease. Also, due to the retrospective study design, we were not able to accurately record all previous DMTs data. A further caveat is the estimated odds ratios, although statistically significant should be interpreted with caution, particularly where all confidence intervals are very wide. The lack of data on spinal cord lesions and atrophy is another major limitation, in knowing the association of spinal lesions with long-term disability.^29^

## Conclusion

Brain atrophy of ICD and TVW early in the cohort of MS could predict progressive disease and disability over 10 years of clinical follow-up, as measured by simple, fast linear measurements which are applicable to clinical practice. The current clinical monitoring relies on T2 lesions development, but this study suggests that simply counting the number of lesions does not have a direct effect on clinical disability 10 years later in the current DMTs treated cohorts. This study also shows that the predictive value of brain lesions and atrophy alone from routine MRI scans may be not enough if used as the sole predictor of outcome. The use of more advanced MRI biomarkers, and especially the integration of these measures in prediction modelling using artificial intelligence platforms, either by way of the use of machine learning (vector) programs with pre-defined features or deep learning techniques, could possibly improve in the future this prediction ability.

## Supplemental Material

Supplemental Material - Predictors of long-term disability in multiple sclerosis patients using routine magnetic resonance imaging data: A 15-year retrospective study Click here for additional data file. Supplemental Material for Predictors of long-term disability in multiple sclerosis patients using routine magnetic resonance imaging data: A 15-year retrospective study by Amjad Altokhis, Abdulmajeed Alotaibi, Paul Morgan, Radu Tanasescu and Nikos Evangelou in The Neuroradiology Journal
