# Supplementary material for: Predictors of long-term disability in multiple sclerosis patients using routine magnetic resonance imaging data: A 15-year retrospective study
Source: Neuroradiol J. 2023 Feb 6;36(5):524–32. doi: 10.1177/19714009221150853 (PMC10569198; doi:10.1177/19714009221150853)
Supplement: Supplemental Material - Predictors of long-term disability in multiple sclerosis patients using routine magnetic resonance imaging data: A 15-year retrospective study [file sj-pdf-1-neu-10.1177_19714009221150853.pdf]

Supplementary

Supplementary 1:

The table illustrates the correlation coefficients, inter-class correlations (ICC) for Inter and intra-rater reliability for all MRI measures.

**Measures**	**Intra-rater**	**Inter-rater**
Lesion counts	0.88 (P < 0.05)	0.80 (P < 0.05)
Lesion volume	0.80 (P < 0.05)	0.83 (P < 0.05)
Third ventricle	0.99 (P < 0.05)	0.86 (P < 0.05)
Corpus Callosum	0.65 (P < 0.05)	0.66 (P < 0.05)
Medulla	0.81 (P < 0.05)	0.64 (P < 0.05)
Inter-caudate distance	0.97 (P < 0.05)	0.70 (P < 0.05)

P<0.05 was set as significant.

Supplementary 2:

Atrophy

Four linear measures were used: TVW[4,32,33], MEDW5,29,30, CCI[36] and ICD[32,37]. TVW was measured as the width of the third ventricle at the midpoint of a line running parallel to the long axis of the ventricle on axial T2-weighted MRI scans. MEDW was measured as the dorsoventral diameter of the medulla on a mid-sagittal image. The level of medullary measurement was determined by the craniocaudal pontine length mirrored caudally from the inferior pontine notch. CCI was obtained on a conventional best mid-sagittal FLAIR image by drawing a straight line at the greatest anteroposterior diameter of CCI and a perpendicular at its midline. Anterior, posterior, and medium segments of CCI were measured and normalized to its greatest anteroposterior diameter. ICD was measured on axial T1-weighted when the frontal horn reached the maximum width. ICD width is the minimum distance between medial borders of the head of the caudate nuclei [32,37]. All measurements were explained below with figures.

1. **Third Ventricle Width**

Width

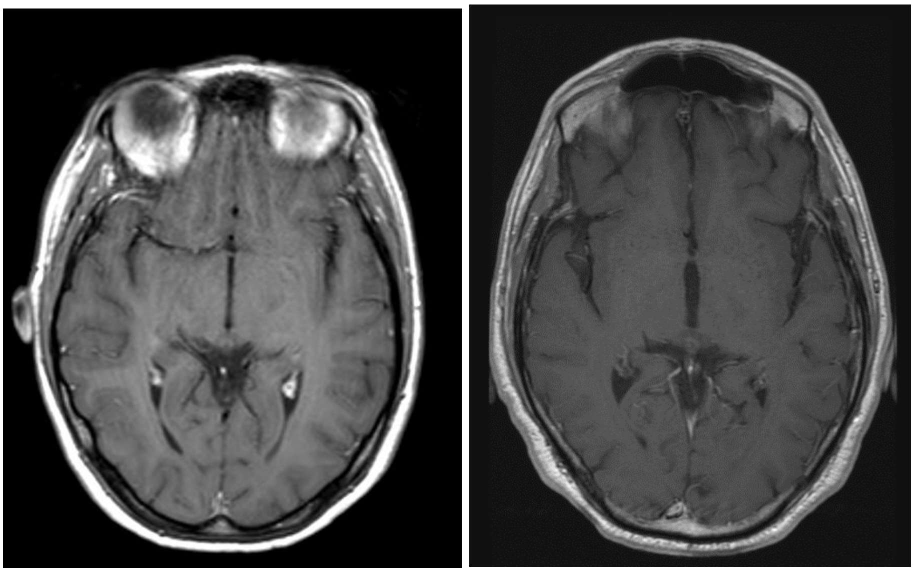

Figure 1. Third Ventricular (TVW) was measured as the

width of the third ventricle at the midpoint (see white arrow) of a line running parallel to the long-axis of the ventricle on axial T_1- weighted MRI scans.

2. Corpus Callosum Index

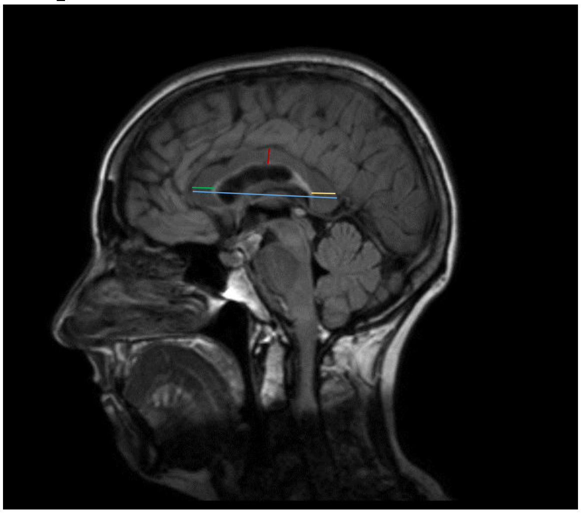

Figure 2. Sagittal FALIR MRI scan.

Corpus Callosum Index (CCI) was obtained on a conventional best mid-sagittal FLAIR image, by drawing a straight line at the greatest anteroposterior diameter of CC and a perpendicular at its midline, owing to points a, b and c. Anterior (green line), posterior (yellow line) and medium (red line) segments of CC were measured and normalized to its greatest anteroposterior diameter (blue line).

$$CCI = \frac{\text{Anterior} + \text{Posterior} + \text{medium}}{\text{Anteroposterior diameter}}$$

3. Medulla Width

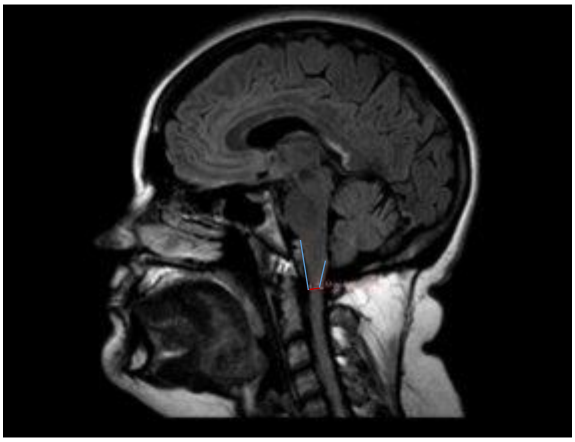

Figure 3. Sagittal FLAIR image and the red line shows the medulla width measure.

- Medulla width (MEDW) was measured as the dorsoventral diameter of the medulla on a mid-sagittal image.
- The level of medullary measurement was determined by the craniocaudal pontine length mirrored caudally from the inferior pontine notch.

4. Inter-caudate distance

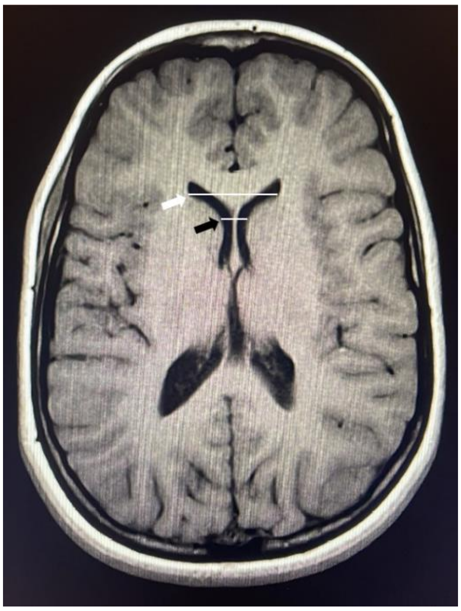

Figure 4. Axial T_{1-} weighted brain MRI, the black arrow shows the ICD measure.

- Inter-caudate distance (ICD) was measured on an axial T_1-weigted image when the frontal horn (white arrow) reached the maximum width.
- ICD width is the minimum distance between medial boarders of the head of the caudate nuclei.

Supplementary 3:

The results from the ordinal regression showed that TVW and ICD at both baseline and follow-up could predict the long-term disability. Meaning an increase of 1 unit at these MRI measurements whether at baseline or follow-up would increase one scale on EDSS at 10 years. All predictors are illustrated in the table below.

Predictors	Odd ratio	95% CI	P-value
BL lesion count	1.01	(0.98-1.04)	0.40
FU lesion count	1.01	(0.98-1.03)	0.37
BL lesion volume	1.04	(0.97-1.12)	0.24
FU lesion volume	1.03	(0.96-1.10)	0.45
BL CC	0.01	(5.08e-6 -77.7)	0.34
FU CC	0.28	(8.29e-5-1010)	0.76
BL Medulla	1.01	(0.73-1.40)	0.95
FU Medulla	0.89	(0.62-1.28)	0.56
BL ICD	1.18	(1.03-1.36)	0.01*

Table. Ordinal regression for the predictors and EDSS

FU ICD	1.14	(1.02-1.28)	0.02*
BL TVW	1.48	(1.11-2.00)	<0.001**
FU TVW	1.29	(1.02-1.63)	0.03*

*Significant at P<0.05, **Significant at P<0.001.
